# Methicillin-resistant *Staphylococcus aureus* emerged long before the introduction of methicillin into clinical practice

**DOI:** 10.1186/s13059-017-1252-9

**Published:** 2017-07-20

**Authors:** Catriona P. Harkins, Bruno Pichon, Michel Doumith, Julian Parkhill, Henrik Westh, Alexander Tomasz, Herminia de Lencastre, Stephen D. Bentley, Angela M. Kearns, Matthew T. G. Holden

**Affiliations:** 1 School of Medicine, University of St Andrews, St Andrews, KY16 9TF UK; 2 School of Medicine, University of Dundee, Dundee, DD1 9SY UK; 3 Antimicrobial Resistance and Healthcare Associated Infections Reference Unit, National Infection Service, Public Health England, Colindale, UK; 4 The Wellcome Trust Sanger Institute, Wellcome Trust Genome Campus, Hinxton, Cambridge, UK; 5 Department of Clinical Microbiology, Hvidovre University Hospital, Hvidovre, Denmark; 6 Institute of Clinical Medicine, University of Copenhagen, Copenhagen, Denmark; 7 Laboratory of Microbiology and Infectious Diseases, The Rockefeller University, New York, USA; 8 Laboratory of Molecular Genetics, Instituto de Tecnologia Química e Biológica António Xavier (ITQB), Universidade Nova de Lisboa, Oeiras, Portugal

**Keywords:** *Staphylococcus aureus*, MRSA, Antibiotic resistance

## Abstract

**Background:**

The spread of drug-resistant bacterial pathogens poses a major threat to global health. It is widely recognised that the widespread use of antibiotics has generated selective pressures that have driven the emergence of resistant strains. Methicillin-resistant *Staphylococcus aureus* (MRSA) was first observed in 1960, less than one year after the introduction of this second generation beta-lactam antibiotic into clinical practice. Epidemiological evidence has always suggested that resistance arose around this period, when the *mecA* gene encoding methicillin resistance carried on an SCC*mec* element, was horizontally transferred to an intrinsically sensitive strain of *S. aureus*.

**Results:**

Whole genome sequencing a collection of the first MRSA isolates allows us to reconstruct the evolutionary history of the archetypal MRSA. We apply Bayesian phylogenetic reconstruction to infer the time point at which this early MRSA lineage arose and when SCC*mec* was acquired. MRSA emerged in the mid-1940s, following the acquisition of an ancestral type I SCC*mec* element, some 14 years before the first therapeutic use of methicillin.

**Conclusions:**

Methicillin use was not the original driving factor in the evolution of MRSA as previously thought. Rather it was the widespread use of first generation beta-lactams such as penicillin in the years prior to the introduction of methicillin, which selected for *S. aureus* strains carrying the *mecA* determinant. Crucially this highlights how new drugs, introduced to circumvent known resistance mechanisms, can be rendered ineffective by unrecognised adaptations in the bacterial population due to the historic selective landscape created by the widespread use of other antibiotics.

**Electronic supplementary material:**

The online version of this article (doi:10.1186/s13059-017-1252-9) contains supplementary material, which is available to authorized users.

## Background

Methicillin-resistant *Staphylococcus aureus* (MRSA) has been identified as one of the major risk pathogens associated with the development of antimicrobial resistance (AMR). The emergence of AMR in *S. aureus* is well documented and the species has proven particularly adept at evolving resistance in the face of new antibiotic challenges. The introduction of penicillin in the 1940s heralded a revolution in the treatment of infectious diseases. However, at the same time as its use was becoming more widespread following advances in the scaling up of production, evidence of penicillin resistance in *S. aureus* was already being uncovered [1].

Methicillin (Celbenin), a semi-synthetic β-lactam, was introduced in the UK in 1959 to circumvent growing penicillin resistance in *S. aureus*, associated with the acquisition of a β-lactamase enzyme, *blaZ* [2]. As a second-generation β-lactam antibiotic, methicillin was insensitive to breakdown by BlaZ. Following the introduction of methicillin into clinical practice in the UK, the Staphylococcal Reference Laboratory in Colindale (London, England) screened *S. aureus* isolates for evidence of resistance to this antibiotic [3]. More than 5000 *S. aureus* strains were assessed in the period between October 1959 and November 1960, and in October 1960 three isolates showing increased minimum inhibitory concentrations (MICs) to the new drug, methicillin, were identified. The isolates originated from the same hospital and shared a common phage type and resistance profile (penicillin, streptomycin, and tetracycline), suggesting that they were related. In the description of these isolates it was noted that methicillin had been used only once previously at this hospital, and that none of the individuals from whom MRSA was isolated had been exposed to the drug. Within 2 years MRSA was being detected elsewhere in Europe, with invasive infections being identified in Denmark [4]. These MRSA isolates from the UK and Denmark in the early 1960s constitute the very first epidemic MRSA clone.

The genetic basis of methicillin resistance in *S. aureus* is associated with carriage of a mobile cassette of genes known as the staphylococcal cassette chromosome *mec* (SCC*mec*) [5]. Within this cassette is the *mecA* gene that is responsible for resistance to β-lactams including methicillin. The product of *mecA* is the peptidoglycan synthesis enzyme penicillin binding protein (PBP) 2a involved in cross-linking of peptidoglycan in the bacterial cell wall [6, 7]. PBP2a has a lower binding affinity for β-lactam antibiotics than the native PBP proteins encoded in the core genome of *S. aureus*. The subsequent combination of reduced penicillin-binding affinity and increased production of PBP2a accounts for the observed resistance to β-lactam antibiotics.

Genetic analyses of the first MRSA by multi-locus sequence typing (MLST) demonstrated that they were sequence type (ST) 250, a lineage belonging to clonal complex (CC) 8 and carried the type I SCC*mec* element [8, 9]. After emerging in the UK, this first epidemic MRSA clone (ST250-MRSA-I) spread across Europe during the 1960s and 70s, but by the late 1980s had become less prevalent and is now rarely reported [9–11]. The single locus variant and close relative of ST250-MRSA-I, ST247-MRSA-I was first detected in Denmark in 1964 [8] and has been more successful, spreading globally and persisting as a source of outbreaks in Europe into the late 1990s [10, 11], but this too has been superseded by more successful contemporary clones [10]. Five decades on since the appearance of the first MRSA, multiple MRSA lineages have emerged which have acquired different variants of SCC*mec* elements.

Epidemiological evidence has always suggested that MRSA arose as a consequence of the introduction of methicillin into clinical practice. Here we have used whole genome sequencing of a collection of 209 of the earliest MRSA isolates recovered in Europe between 1960 and 1989 to reconstruct the evolutionary history of methicillin resistance. Using Bayesian phylogenetic reconstruction we have identified the likely time point at which this early lineage arose and also predicted the time around which SCC*mec* was acquired.

## Results

### Early MRSA belong to a diverse clone

Preserved in the culture collection of the Staphylococcal Reference Laboratory at Public Health England are representatives of the very first MRSA identified. These original isolates have been preserved as freeze-dried cultures, and have not been repeatedly passaged over the years. One hundred and eighty eight isolates that represented the earliest MRSA were recovered from the ampoules and their genomes sequenced (Additional file 1: Table S1). All the isolates belonged to CC8 MRSA and were originally isolated between 1960 and the late 1970s, and included eight isolates from the original study describing MRSA in 1961 [3]. In addition, 21 CC8 MRSA isolated between 1964 and 1989 in Denmark [8, 11] were sequenced, as representatives of the earliest MRSA detected elsewhere in Europe. We also included early methicillin-sensitive isolates of ST250 or ST247 (*n* = 11); however, only a limited number of these were found in the reference laboratory collection.

Analysis of the MLST of the isolates identified two main groups, ST250 (*n* = 126) and a single locus variant (SLV), ST247 (*n* = 78), plus two novel SLVs of ST247 (*n* = 4) (Additional file 1: Table S1). A supplementary isolate from the Public Health England collection was included to provide an outgroup for the analysis; RH12000692_7401696 is an MRSA which was collected in 1967 and is a triple locus variant of ST250 (Additional file 1: Table S1).

**Fig. 1:**
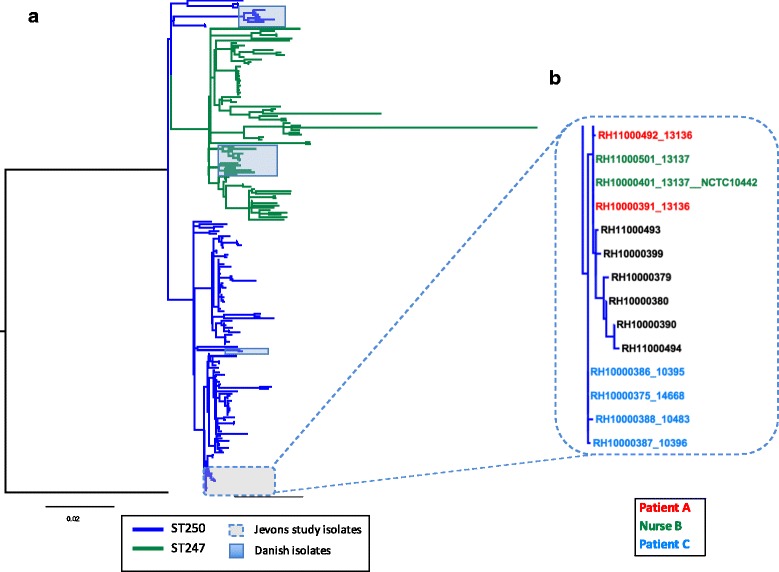
Population structure of historic MRSA isolates. **a** Maximum likelihood tree of historic MRSA isolates. The tree was built using a maximum likelihood method using SNPs from the core genome of 209 isolates. Included in the phylogeny is the COL reference isolate to which the sequence reads were mapped. The tree is rooted with RH12000692_7401696 as an outgroup; this is a CC8 isolate and is a triple locus variant of ST250. Tree branches are coloured according to their ancestral sequence type population; *blue branches* indicate the ST250 population and *green branches* the ST247 population. Isolates from Denmark are highlighted in *blue shading* and isolates described in the Jevons study are outlined in the *dashed box*, and a zoomed in view of the phylogeny is displayed in **b**. The coloured branch labels indicate the three individuals who supplied the original isolates in the Jevons study

The *S. aureus* isolate COL, a representative member of this early MRSA lineage first identified in the 1960s [12], had previously been fully sequenced, and the chromosome was used as a reference for mapping. Following exclusion of mobile genetic elements (MGEs) and predicted recombination events in the collection, a total of 4220 SNPs were identified and used to construct a phylogeny (Fig. 1a). The population framework revealed a diverse population structure containing several distinct clades. The mapping of the ST information on to the phylogeny reveals that the ST250 population is basal to the ST247, suggesting that ST247 emerged from ST250, which is consistent with the epidemiological evidence, and supports the hypothesis that this pandemic multidrug-resistant MRSA clone emerged out of the ancestral MRSA genotype [8, 9].

Highlighted in the expanded view (Fig. 1b) are the isolates from the Jevons study, derived from three individuals at the same hospital in the South London area between July and November 1960 [3]. The isolation source and resistance profiles of these isolates are shown in Additional file 2: Table S2. These isolates are genetically very closely related, differing by seven SNPs only. Present within this cluster are additional isolates from the Public Health England collection originating between 1960 and 1961. Full epidemiological data are not available for these, but two of these isolates were identified in the same region as the hospital where the original Jevons study isolates originated. The genetic distance between isolates and their phylogenetic relationships suggests there was transmission within the hospital between patients A and C and nurse B, and that they were also transmitted beyond the hospital as part of a local outbreak.

**Fig. 2:**
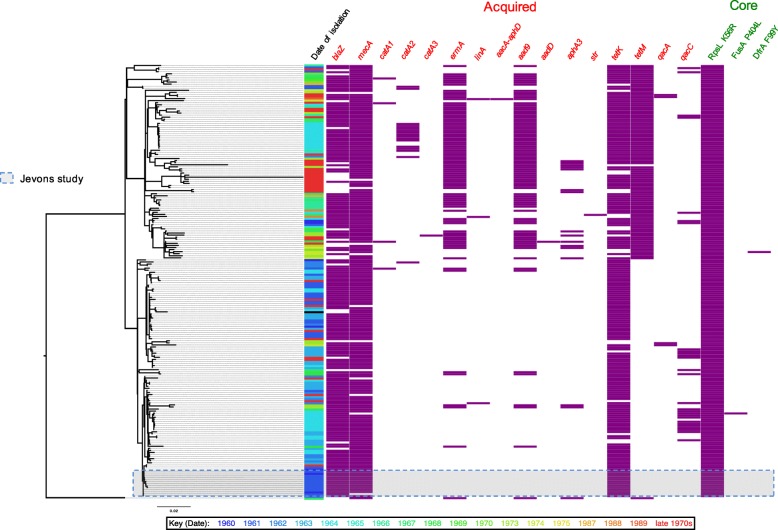
Distribution of antibiotic resistance determinants in the archetypal MRSA clone. A maximum likelihood tree of historic MRSA isolates (*n* = 209) plus the COL reference is displayed on the *left*, and the panels on the *right* indicate dates of isolation (coloured according to year; see key below for years), and the presence (*purple boxes*) and absence (*white space*) of genetic determinants responsible for antibiotic resistance in the genomes of the isolates. The identity of genetic determinants are listed at the *top* and divided into acquired genes (*red text*; *left hand side*), and core mutations (*green text*; *right hand side*). The antibiotics linked to the genetic determinants for the acquired genes are: β-lactams, *blaZ* and *mecA*; chloramphenicol, *catA*1, *catA*2, and *catA*3; erythromycin, *ermA*; clindamycin, *linA*; aminoglycosides, *aacA*-*aphD*, *aad9*, *aadD*, *aph*3A, and *str*; tetracycline, *tetM* and *tetK*; disinfectants, *qacA* and *qacC*. And for the core gene mutations are: streptomycin substitution of arginine for a lysine at residue 56 (K56R) of the ribosomal protein *rpsL*; fusidic acid, substitution of a proline for a leucine at residue 406 (P404L) of the transcription elongation factor *fusA*; trimethoprim, substitution of an tyrosine for a phenylalanine at residue 99 (F99Y) of the dihydrofolate reductase *dfrA*. Sixteen isolates lacked complete type I SCC*mec* elements, 4 of which contained internal deletions in the SCC*mec* element but retained the *mecA* gene

Although all of the Jevons isolates are confined to a single clade, other isolates from the early 1960s are distributed throughout the entire phylogeny (Fig. 2). This suggests that the earliest MRSA circulating in the UK were not from a single recently emerged clone, but were representatives of an established population. In addition to the UK isolates, there were 21 from Denmark, which represent the earliest MRSA detected outside the UK. These derive from 1964 onwards, and include the youngest isolates within the collection from the late 1980s. The Danish isolates are found in three clusters distributed throughout the phylogeny (Fig. 1a), suggesting that, like the early UK MRSA, they originated from an established and diverse population.

### Genetic basis of resistance to methicillin and other antibiotics in the archetypal MRSA population

Previous studies have shown that the archaic MRSA clone carried a type I SCC*mec* element, which was the first type of this MGE family to be classified [5, 13]. Notably, the description of the type I element was based upon the SCC*mec* derived from *S. aureus* strain NCTC10442 identified in the 1960 Jevons study (Fig. 1b; Additional file 2: Table S2) [13]. The type I element carries *mecA* as its only resistance gene in combination with a truncated gene encoding the MecRI regulatory proteins (together known as a class B *mec* gene complex) with type 1 chromosomal recombinases (*ccrA1* and *ccrB1*). The original description of SCC*mec* type I identified the presence of a frameshift mutation in *ccrB1* which disrupts the translation of this site-specific recombinase [13]; the mutation occurs after codon 321 and is caused by a single base deletion in a poly-A hexamer resulting in a pentamer sequence. In the collection, 193 of the isolates contained intact SCC*mec* elements carrying the *mecA* gene (Fig. 2). Of these, 192 were SCC*mec* type I elements, all of which contained the pentamer sequence and the same frameshift mutation in *ccrB1* as the NCTC10442 reference. The only non-type I element identified in the collection was in the outgroup isolate RH12000692_7401696, which contained a type IVh SCC*mec* element. The remaining 16 isolates lacking complete SCC*mec* elements were distributed throughout the phylogeny, suggesting that these represent methicillin-sensitive *S. aureus* (MSSA) arising from the loss of the type I SCC*mec* element, rather than forming an ancestral MSSA population.

In addition to methicillin resistance, the first MRSA described were also resistant to penicillin, streptomycin and tetracycline [3]. Analysis of the genomes of these isolates identified *blaZ* and *tetK* genes conferring resistance to penicillin and tetracycline, respectively, but failed to identify the *str, aadE* or *aad9* genes associated with streptomycin resistance in *S. aureus*. In the absence of an acquired resistance gene, the core genome was examined for mutations potentially responsible for resistance to streptomycin. In *Mycobacterium tuberculosis*, mutations in the ribosomal protein RpsL were shown to confer streptomycin resistance, including the substitution of an arginine for a lysine residue at residue 43 [14]. Alignment of the *M. tuberculosis* and *S. aureus* sequences revealed that RpsL in the Jevons isolates contained an arginine in the equivalent position, residue 56. Comparison with RpsL sequences in the public sequence databases showed that in *S. aureus* the frequent amino acid residue at position 56 was lysine. Examining the whole collection, all but one of the sequenced isolates contained the arginine residue at position 56, the exception being the outgroup isolate RH12000692_7401696 (Fig. 2). This demonstrates that the non-synonymous substitution resulting in an arginine for a lysine residue at residue 56 (K56R) occurred most likely very early during emergence of the archetypal MRSA population.

*In silico* analysis of the resistomes of the isolates revealed genetic resistance determinants to numerous other antibiotics, including penicillin (*blaZ*), erythromycin (*ermA* and *linA*), kanamycin (*aadD*), gentamicin and kanamycin (*aacA*-*aphD*), spectinomycin and streptomycin (*aad9*), and chloramphenicol (*catA1*, *catA2* and *catA3*), fusidic acid (*fusA* P404L) and trimethoprim (*dfrA* F99Y), as well as genes associated with decreased susceptibility to disinfectants (*qacA* and *qacC*). The frequency and widespread dispersal of these determinants reveal the strong selective pressure exerted by antibiotics on the archetypal MRSA clone over an extensive period. Examining their distribution in the context of the phylogeny shows that some of these traits have been co-acquired (Fig. 2), such as *ermA* and *aad9*, which are carried on Tn*554*, and that these acquisition events can be mapped on to the phylogeny [15].

### Evolution and emergence of methicillin resistance

To determine if the methicillin resistance emerged once or multiple times in the archetypal MRSA population, we examined the variation within the SCC*mec* type I elements. In total, 194 variant sites were identified in 192 elements present in the collection. Analysis of the distribution of the variation within the elements suggested that some could be attributed to homologous recombination. Two regions contained the majority of the variation: 124 SNP sites were identified in the gene encoding the LPxTG surface protein *pls*, and 31 SNP sites within a 549-bp intergenic region between a hypothetical protein (SACOL0030) and a glycerophosphoryl diester phosphodiesterase (SACOL0031). Excluding these predicted recombination regions, 39 core variants sites across 28.6 kb distinguished the 192 elements, with half of the isolates (n = 96) carrying an identical element. The maximum SNP distance distinguishing any two elements was eight SNPs, and phylogenetic analysis revealed that the elements present in the historic MRSA clone were closely related (Additional file 3: Figure S1) and shared a common evolutionary origin.

**Fig. 3:**
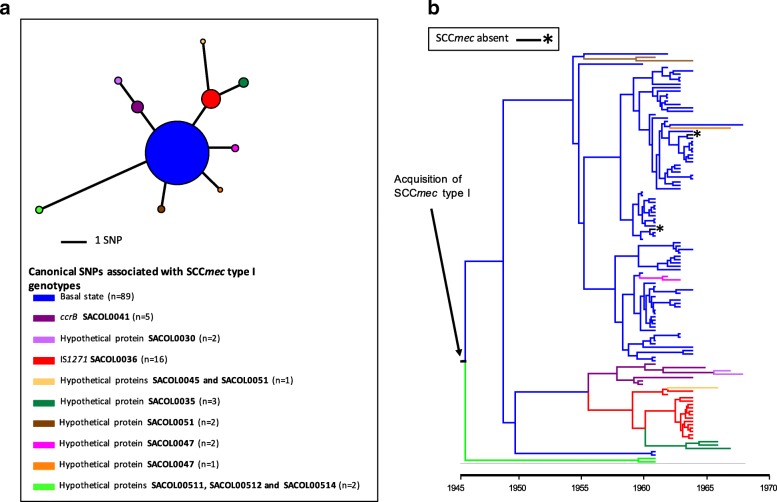
Diversity and distribution of SCC*mec* type I elements in the archetypal MRSA population. **a** Parsimonious minimal spanning tree of SCC*mec* type I elements present in the archetypal MRSA isolates present in the clade credibility tree in **b**. The tree is built with core SNPs identified in the SCC*mec* type I elements, and excludes SNP in the *pls* gene that were predicted to have arisen by recombination. In total, ten genotypes were observed, and the genetic events that distinguish each genotype from the founder genotype are indicated. The tree is centred on the majority genotype inferred as the founder population, and colour-coded according to their genotype. *Black asterisks* indicate isolates that lack the type I SCC*me*c element. The sizes of the *circles* illustrate the relative sizes of the genotype populations. The *key* below the tree describes the canonical SNPs differentiating SCC*mec* type I genotypes and the number of variants with that genotype. **b** Maximum clade credibility tree of the archetypal MRSA clone population based on BEAST analysis. Tips of the tree are constrained by isolation dates; the time scale is shown *below* the tree. The tree is built with core genome SNPs from a subset of the total collection’s isolates (n = 122), which had precise dates of isolation, and whose origins could be linked back to the original submission documentation. The branches of the tree are coloured according to the genotype of the SCC*mec* type I element present in that strain (illustrated in **a**). Internal branches are coloured according to parsimonious reconstruction of the predicted genotype. Where terminal branches are *black* and highlighted by a *black asterisk*, this indicates the absence of an SCC*mec* element, which is predicted to reflect loss of the element. An *arrow* indicates the point in the phylogenetic reconstruction where an ancestral type I SCC*mec* element was acquired. The root of the tree corresponds to the basal node of the ST250/ST247 population in Fig. 1 rooted by the RH12000692_7401696 outgroup. From the analysis the estimated mutation rate of population is 1.8 × 10^−6^ SNPs/site/year. This substitution rate falls within the reported ranges of multiple successful *S. aureus* lineages [31] and therefore it is unlikely likely that long-term storage of the isolates has created any temporal artefacts

Our analysis of the evolutionary events surrounding the emergence of methicillin resistance in the archetypal MRSA lineage focused on a subset of 122 isolates that had precise dates and places of origin which could be linked to original submission records (Additional file 2: Table S1). This enabled us to generate a robust Bayesian phylogeny and temporal calibration. Examining the distribution of the type I SCC*mec* variants (Fig. 3a) within the context of a core genome phylogeny generated with BEAST (Fig. 3b) revealed congruence between the phylogenetic relationships of the two. All of the canonical SNPs associated with the SCC*mec* genotypes could be singularly mapped onto nodes of the core phylogeny, suggesting that the variation observed in the SCC*mec* elements had occurred during expansion of the ST250 and ST247 populations. On the basis of this, we propose that a type I SCC*mec* element was acquired once in a single primordial development of methicillin resistance (Fig. 3b) that could be dated back to the emergence of this clone.

In our Bayesian phylogenetic analysis of the core genome SNPs we utilized a range of population and clock model combinations. The combination of an exponential population and relaxed-log normal clock model was found to be the best fit to our data based on Bayes factors using the harmonic mean estimator. This indicated the time to most recent common ancestor (TMRCA) of the ST250/ST247 population was 1946 (95% highest posterior density (HPD) 1938–1952) (Additional file 3: Figure S2), and therefore the time of acquisition of SCC*mec* was likely around, or before, this date. Notably, the TMRCA of the type I SCC*mec* elements in these isolates based on a linear regression of a core SNP phylogeny was predicted to be early 1941 (Additional file 3: Figure S3).

**Fig. 4:**
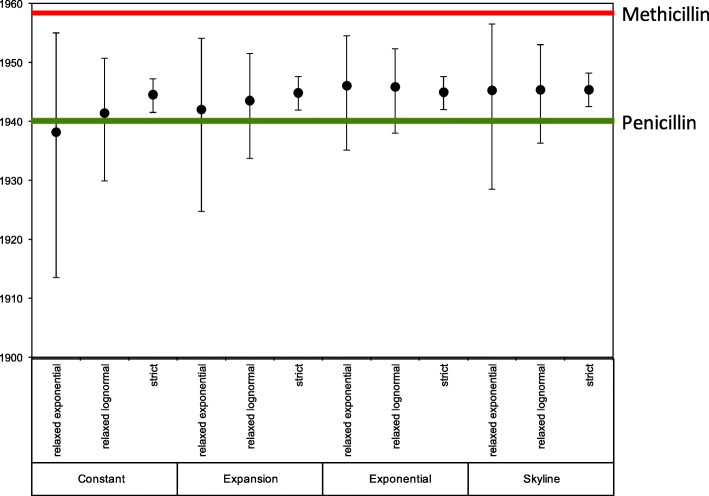
The time to most recent common ancestor (TMRCA) of the archetypal MRSA isolates under various combinations of clock and population model in BEAST. Plots showing mean (*dots*) TMRCA and 95% highest posterior density for the TMRCA are indicated. The dates of introduction of penicillin and methicillin into clinical use in the UK are indicated with *green* and *red lines*, respectively

To ensure that the Bayesian result was not an artefact of the clock or population models used in the analysis, we calculated the TMRCA for a range of model combinations and found that our chosen model exhibited a predicted TMRCA that was encompassed by the 95% HPDs of all other model combinations (Fig. 4).

## Discussion

This historic collection provides unique insights into of the evolution of the first MRSA lineage. Preserved for decades in their original freeze-dried state, this large collection of strains representing the earliest MRSA clone has allowed us to reconstruct the evolutionary events leading to the emergence of MRSA. Using whole genome sequencing we have been able to shed light on the time when SCC*mec* first entered into *S. aureus*, and also to estimate how many times this is likely to have happened in the archaic MRSA population.

The origins of SCC*mec* almost certainly lie in coagulase-negative staphylococci (CoNS) [16]. *S. aureus* belonging to the ST250 background appear to have been the first recipient in the transfer from CoNS, but whether the element entered the ST250 population on multiple occasions, or as a single isolated event with subsequent propagation through the population, has never been definitively resolved. A single entry of *mecA* followed by its evolution within the recipient background has been suggested [17]. In order to clarify this we examined the variation present within the SCC*mec* elements in isolates throughout the population. The variation seen within SCC*mec* is predominantly in the *pls* gene, which has been described before [18]. Functionality of this 230 kDa cell wall-anchored (CWA) protein remains unclear, but its expression has been shown to reduce adhesion to host proteins as well as decrease invasiveness [19]. This LPxTG surface protein has a highly repetitive D/S-rich structure, making it a target for homologous recombination. As noted in other lineages the CWA proteins are subject to diversifying selection and exhibit diversity between and within *S. aureus* lineages [20, 21]. Removal of this variation reveals that the evolutionary history of the SCC*mec* elements was congruent with that of the strains carrying them, which points towards a single acquisition, rather than multiple or recurrent horizontal transmissions. Supporting this hypothesis is the observation of a mutation in *ccrB1* gene of the SCC*mec* type I element. The recombinase genes are required for both integration and excision from the chromosome. Specifically, CcrB is required for excision and the mutation present within this NCTCT10442 type I SCC*mec* element is believed to produce a non-functioning recombinase [22, 23]. Given that all the isolates in this collection have this frameshift mutation, this strongly supports the conclusions of the phylogenetic analysis, namely that a type I SCC*mec* was acquired once in the ST250 background, and then became fixed in the population due to defective recombinase apparatus that precluded excision.

One of the questions we sought to address in this study was what were the temporal events surrounding the emergence of MRSA. With the first reports of MRSA occurring only after introduction of methicillin in the UK in 1959 and Denmark in 1964 it seemed reasonable to conclude that resistance arose after the first clinical use of the drug, and resistance therefore developed in *S. aureus* as an adaptive response following exposure to the antibiotic. However, the results presented in this communication are not consistent with this conclusion, since the gene bestowing methicillin resistance was likely to have been acquired in the mid-1940s. It was during this period that β-lactamase-mediated penicillin resistance was becoming widespread among clinical isolates of *S. aureus*. Within 4 years of the introduction of penicillin for the treatment of staphylococcal infections, the first penicillin-resistant *S. aureus* were being described in 1944 [1]. In the years that followed the frequency of resistance in clinical isolates climbed steadily, such that by the time methicillin was introduced into clinical practice in 1960, resistance rates of 80% were common [24, 25].

Whilst the main genetic determinant associated with penicillin resistance in *S. aureus* is *blaZ*, *mecA* also encodes penicillin resistance via a different mechanism involving an alternative penicillin-binding protein, PBP2a [6, 26]. In the sequenced collection *blaZ* is widely distributed, albeit at a lower frequency than *mecA* (85.2% of isolates carry the *blaZ* gene in comparison to 95.2% for *mecA*), suggesting a selective advantage to possessing two distinct β-lactam resistance mechanisms. Based on the temporal calibration of the acquisition of *mecA*, it appears likely that methicillin resistance in *S. aureus* evolved long before this new β-lactam antibiotic was introduced. Thus, it was the widespread use of penicillin, rather than methicillin, that was the driver for the emergence in the archaic MRSA clone.

Beyond β-lactams our analysis uncovered evidence for the strong selective impact that a number of different antibiotics have had on the evolution of the archaic MRSA clone. Several of the antibiotics, such as tetracycline, are prescribed in far lower amounts today in human medicine than in the 1950s and 1960s, and resistance to these antibiotics in contemporary *S. aureus* from humans is relatively rare, which contrasts with the archaic MRSA population, in which the distribution of tetracycline resistance determinants was widespread (Fig. 2; 96% of isolates contained *tetK* or/and *tetM*) [27]. In a prescient study examining the antibiotic consumption and rates of resistance in a hospital in the US in the 1950s, Bauer et al. provided evidence of a correlation between the two, where increasing usage of tetracycline was associated with an increase in the frequency of tetracycline resistance in isolates from inpatients [25].

In addition to methicillin and tetracycline resistances, a key phenotypic marker of the archaic MRSA clone was non-susceptibility to streptomycin. In our analysis we identified a mutation predicted to confer streptomycin resistance occurring on the same branch of the tree in which we mapped the acquisition of the SCC*mec* element. This finding suggests that methicillin and streptomycin resistance both emerged in the archetypal MRSA progenitor population around the same time. Discovered in the early 1940s, streptomycin was demonstrated to have activity against Gram-positive pathogens, and was used in the UK in 1947 during the first ever randomized clinical trials studying the efficacy of streptomycin in the treatment of pulmonary tuberculosis [28, 29]. It therefore appears that the first MRSA clone emerged, and developed resistance to two of the earliest antibiotics—streptomycin and penicillin—almost immediately after the *S. aureus* population would have been first exposed to them.

At the time of its discovery, the incidence of MRSA in the general population is likely to have been very low. This is demonstrated by the fact that screening of over 5000 samples at Public Health England yielded only three methicillin-resistant isolates. Therefore, it is likely that when methicillin was introduced to circumvent penicillin resistance in *S. aureus*, it did not select for emergence of MRSA at that time, but instead provided the selective pressure, which drove the nosocomial spread of a pre-existing variant, at a time when infection control measures in UK hospitals were limited.

## Conclusions

This study highlights the unintended consequences of widespread antibiotic use, and how when new drugs are introduced to bypass known resistance mechanisms, they may already be rendered ineffective due to unrecognised adaptations accrued in response to prior selective pressures exerted by other antibiotics. This remains one of the many challenges in tackling the growing problem of AMR and serves to emphasise the importance of continual surveillance of pathogen populations for evidence of emerging adaptations and resistance patterns in the context of prescribing practice.

## Methods

### Bacterial isolates

Two hundred and nine isolates derived from the culture collections of *Staphylococcus aureus* reference laboratory, Public Health England, and isolates originating from the Statens Serum Institute collected and analysed by Profs Tomasz, Westh and de Lencastre. These correspond to a collection of MRSA and MSSA isolates collected between 1960 and the late 1980s in the UK and Denmark. Isolates from the Public Health England collection were all retrieved from the original freeze-dried cultures put down in the 1960s. All the Statens Serum Institute isolates were kept in a lyophilized state until the late 1990s, when they were opened, sub-cultured, and then stored at –80 °C. A record of the number of sub-cultures prior to freeze-drying, or post-isolate recovery, was not available, but it is known that the isolates were not repeatedly sub-cultured over the last 50 years.

One hundred and eighty eight isolates preserved as freeze-dried cultures in the Health Protection England (HPA) Staphylococcal Reference Laboratory were resurrected and grown on solid media. Prior to the start of this study the Reference Laboratory sequence typed all isolates from 1960 and 1961 using standard MLST techniques [30] and identified that the isolates belonged to CC8 and were either ST250 or ST247.

Twenty-one CC8 MRSA isolated in Denmark between 1964 and 1989 were also included in this study. These isolates originating from the Statens Serum Institute and had been previously sequence typed using standard MLST techniques [30]. All isolates in this study were subsequently sequence typed from their whole genome sequence data (see below).

### Genomic library preparation and sequencing

Genomic DNA was isolated using the Qiagen QIAcube system according to the manufacturer’s protocol.

We prepared sequencing libraries from 500 ng of DNA extracted from each MRSA isolate as previously described, with amplification using Kapa Hifi polymerase (Kapa Biosystems, Woburn, MA, USA) [31]. Tagged DNA libraries were created using a method adapted from a standard Illumina Indexing protocol, as described previously [31]. Whole genome sequencing was performed on the Illumina HiSeq 2000 platform with 100-bp paired-end reads. The Illumina sequence data have been submitted to the European Nucleotide Archive (ENA) and the accession numbers are provided in Additional file 1: Table S1.

### Bioinformatic and phylogenetic analysis

The sequence reads for each representative isolate (*n* = 209) were aligned against the reference genome of the MRSA *S. aureus* COL (accession number CP000046) [32] using SMALT (version 0.7.4; http://www.sanger.ac.uk/science/tools/smalt-0) and SNPs (single nucleotide polymorphisms) and indels (insertions/deletions) identified as described previously [31]. Mobile genetic elements (MGEs) were identified in the COL reference chromosome by comparison with other *S. aureus* chromosomes, where BLASTN (version 1.4) pairwise comparison were visualized in ACT (version 13.0.0) [33]. Regions of recombination within core genome and SCC*mec* element alignments were identified with Gubbins using the default parameters (version 1.4.10; http://github.com/sanger-pathogens/Gubbins) [34]. Phylogenetic reconstruction using core SNPs was performed with RAxML (version 8.2.8), using a GTR model with a gamma correction for among site rate variation [35]. Regions of high-SNP density corresponding to putative regions of recombination and those SNPs associated with horizontal gene transfer were excluded. Assembly of all genomes was performed using a high throughput assembly method [36].

In order to investigate if the genomic data contained evidence of a temporal signal we used root to tip linear regression using Path-O-Gen (version 1.4; http://tree.bio.ed.ac.uk/software/tempest/; Additional file 3: Figure S4). A core alignment for 122 isolates for which precise dates of isolation were available was used. MGEs and regions of predicted recombination along with homoplastic SNPs within these isolates were then also excluded. To estimate evolutionary rates and time to most common recent ancestor (TMRCA) Bayesian phylogenetic reconstruction was performed using BEAST (version 1.7.4) [37]. A GTR model with a gamma correction for among-site rate variation was used, and all combinations of strict, relaxed lognormal, and relaxed exponential clock models and constant, exponential, expansion, and skyline population models were evaluated. For each, three independent chains were run for 100 million generations, sampling every ten generations. On completion each model was checked for convergence, both by checking effective sample size (ESS) values were greater than 200 for key parameters, and by checking independent runs had converged on similar results. Models were compared for their fit to the data using Bayes factors based on the harmonic mean estimator as calculated by the program Tracer (version 1.4) from the BEAST package. A burn-in of ten million states was removed from each of the three independent runs of this model before combining the results from those runs with the logcombiner program from the BEAST package.

A previously described database of sequences of known resistance determinant genes, both horizontally acquired and core, was utilized as a resistome database (Additional file 4: Table S3) [27, 38]. Fastq files from the 209 isolates were mapped to the resistome database with SRST2 (version 0.1.8) using the default settings [39]. SNPs in chromosomally encoded genes previously identified as being associated with antimicrobial resistance were then manually inspected to confirm the variation.

The multilocus sequence type (MLST) of isolates was predicted using SRST2 (version 0.1.8) [39].

## Additional files

Additional file 1: Table S1. Isolate metadata. The table contains information about the origins, mapping and assembly statistics, and genotype information of all the isolates used in the study. For the origins of the isolates, the year or decade of isolation, along with place and country of isolation are provided where known. Isolates designated London originated from multiple hospitals (hospitals 1–5), and these are indicated. Isolates that were described in the original description by P. Jevons, published in the BMJ in 1961 are highlighted. Summary information for the *de novo* assembly and reference mapping of each isolate’s whole genome sequence data is provided, as is the ENA accession number for fastq data. The sequence type (ST) and allele type are provided, and single locus variants (SLV) or a triple locus variant (TLV) are marked. The isolates used in the temporal analysis presented in Figs. 3b and 4 are marked. (XLSX 90 kb)

Additional file 2: Table S2. Isolates from the original description of MRSA. Minimum inhibitory concentration (MIC) to celbenin (methicillin) derived from the original description by P. Jevons, published in the *BMJ* in 1961. MIC values represent the variation noted between colonies. Expected range of sensitivity to celbenin in coagulase-positive staphylococci 1.25–2.5 μg/ml. Acquired antibiotic resistance genes and core resistance mutations identified in the genomes are indicated. (PDF 42 kb)

Additional file 3: Figure S1. Maximum likelihood tree of SCC*mec* type I elements in historic MRSA isolates. **Figure S2.** Posterior support of maximum clade credibility trees of the historic MRSA population based on BEAST analysis (as illustrated in Fig. 3b). **Figure S3.** Linear regression of the root-to-tip distances of historic MRSA SCC*mec* type I elements. **Figure S4.** Linear regression of the root-to-tip distances of the archetypal MRSA clone population used for BEAST analysis. (PDF 302 kb)

Additional file 4: Table S3. Resistome antibiotic determinants. Updated resistome database used for the *in silico* identification of antibiotic-resistant determinants based on the database previous described by Aanensen et al., 2016. Included in the database are antibiotics and associated acquired genes and core gene mutations. Accession numbers for the acquired genes and protein sequences are provided, as well as amino acid substitution information for the core genes. (PDF 80 kb)

## Abbreviations

AMR: Antimicrobial resistance
CC: Clonal complex
CoNS: Coagulase negative staphylococci
CWA: Cell wall-anchored
ENA: European Nucleotide Archive
HPD: Highest posterior density
MGE: Mobile genetic element
MIC: Minimum inhibitory concentration
MLST: Multi-locus sequence typing
MRSA: Methicillin-resistant *Staphylococcus aureus*
MSSA: Methicillin-sensitive *Staphylococcus aureus*
PBP: Penicillin binding protein
SCC*mec*: Staphylococcal cassette chromosome *mec*
SLV: Single locus variant
SNP: Single nucleotide polymorphism
ST: Sequence type
TMRCA: Time to most recent common ancestor
