# Supplementary material for: Methicillin-resistant Staphylococcus aureus emerged long before the introduction of methicillin into clinical practice
Source: Genome Biol. 2017 Jul 20;18:130. doi: 10.1186/s13059-017-1252-9 (PMC5517843; doi:10.1186/s13059-017-1252-9)
Supplement: Supplementary file 2 — Isolates from the original description of MRSA. Minimum inhibitory concentration (MIC) to celbenin (methicillin) derived from the original description by P. Jevons, published in the BMJ in 1961. MIC values represent the variation noted between colonies. Expected range of sensitivity to celbenin in coagulase-positive staphylococci 1.25–2.5 μg/ml. Acquired antibiotic resistance genes and core resistance mutations identified in the genomes are indicated. (PDF 42 kb) [file 13059_2017_1252_MOESM2_ESM.pdf]

Jevons Strain ID	PHE ID	Celbenin MIC (ug/ml)	Source	Date of Isolation	Sequence type	Resistance determinants present
13137	RH11000501	12.5-25	Nurse B	2/10/1960	250	*blaZ* *mecA* *tetK* RpsL(K56R)
13137	RH11000401 (NCTC 10442)	12.5-25	Nurse B	2/10/1960	250	*blaZ* *mecA* *tetK* RpsL(K56R)
13136	RH10000391	6.5-12.5	Patient A	2/10/1960	250	*blaZ* *mecA* *tetK* RpsL(K56R)
13136	RH11000492	6.5-12.5	Patient A	2/10/1960	250	*blaZ* *mecA* *tetK* RpsL(K56R)
10395	RH10000386	6.25	Patient C	21/07/1960	250	*blaZ* *mecA* *tetK* RpsL(K56R)
10396	RH10000387	3.125	Patient C	05/07/1960	250	*blaZ* *mecA* *tetK* RpsL(K56R)
14083	RH10000388	1.6	Patient C	28/10/1960	250	*blaZ* *tetK* RpsL(K56R)
14668	RH10000375	25	Patient C	08/11/1960	250	*blaZ* *mecA* *tetK* RpsL(K56R)
