# Supplementary material for: Methicillin-resistant Staphylococcus aureus emerged long before the introduction of methicillin into clinical practice
Source: Genome Biol. 2017 Jul 20;18:130. doi: 10.1186/s13059-017-1252-9 (PMC5517843; doi:10.1186/s13059-017-1252-9)
Supplement: Supplementary file 3 — Maximum likelihood tree of SCCmec type I elements in historic MRSA isolates. Figure S2. Posterior support of maximum clade credibility trees of the historic MRSA population based on BEAST analysis (as illustrated in Fig. 3b). Figure S3. Linear regression of the root-to-tip distances of historic MRSA SCCmec type I elements. Figure S4. Linear regression of the root-to-tip distances of the archetypal MRSA clone population used for BEAST analysis. (PDF 302 kb) [file 13059_2017_1252_MOESM3_ESM.pdf]

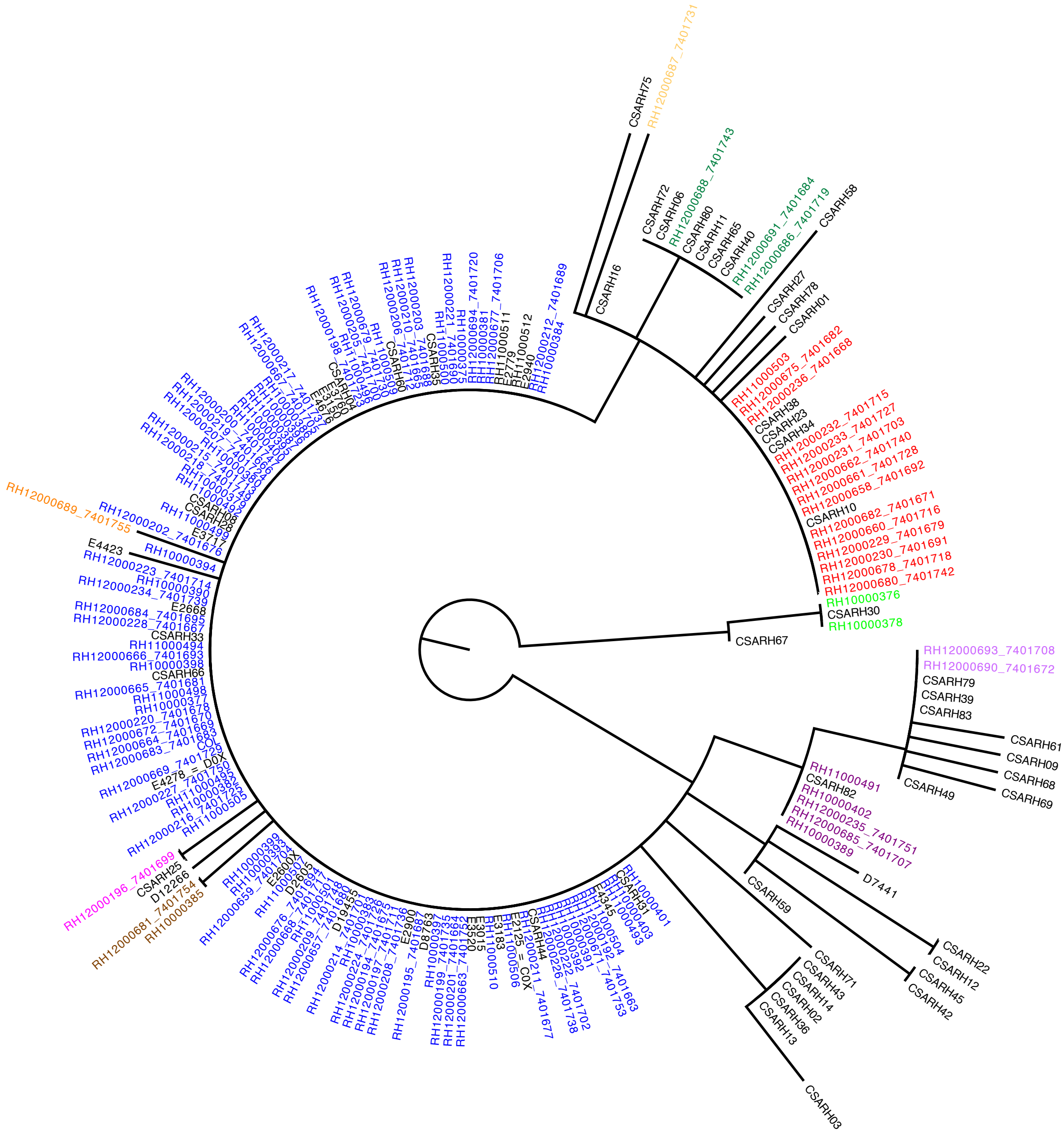

Additional file 3: Figure S1

Maximum likelihood tree of SCC*mec* type I elements in historic MRSA isolates. The tree was constructed using variation in 38 SNP core sites identified in 192 isolates. The coloured branch labels indicate the isolates used in the parsimonious minimal spanning tree of SCC*mec* type I elements illustrated in Figure 3A and the temporal analysis illustrated in Figure 3B (also included in Supplementary Figures 1, 2 and 3), and are colour coded according to the genotypes displayed in this figure. The isolates not included in the temporal analysis are in indicated in black text.

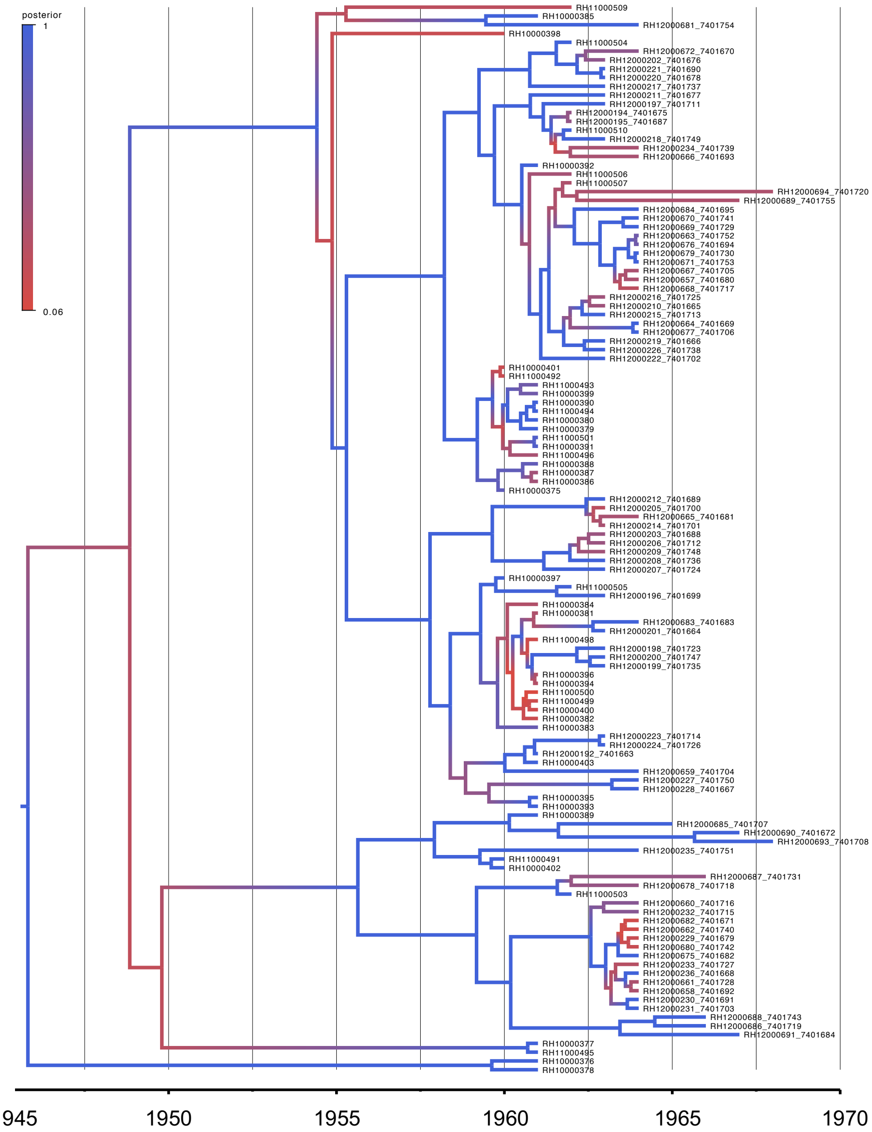

Additional file 4: Figure S2

Posterior support of maximum clade credibility trees of the historic MRSA population based on BEAST analysis (as illustrated in Figure 3B).

Internal branches are colored according to their posterior support (see figure for key).

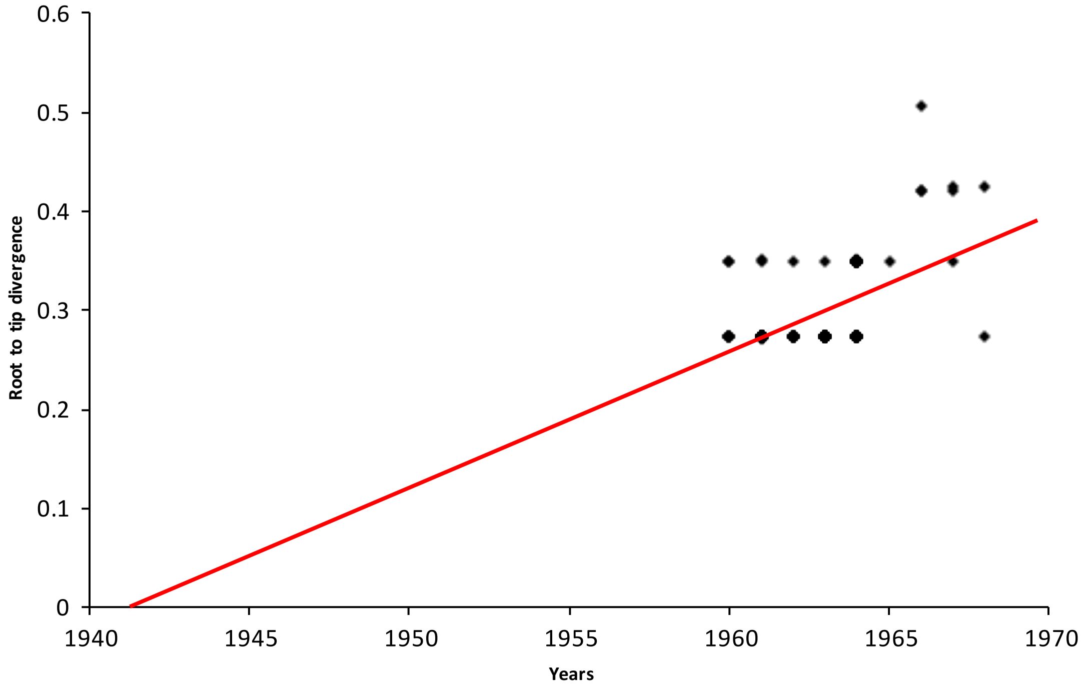

Additional file 5: Figure S3
Linear regression of the root-to-tip distances of historic MRSA SCC*mec* type I elements.
The isolates used (n=122) are those indicated in the Additional file 6: Figure S4 and used for the BEAST analysis (Figure 3B). The analysis was carried out using Path-O-Gen v1.4 (http://tree.bio.ed.ac.uk/software/pathogen/) with a best-fit root from the maximum likelihood tree and the dates of isolation. The plot contains straight-line best fit of the root-to-tip divergence for each of the isolates, with a correlation coefficient of 0.5408 and an R^2 of 0.2925. The time to most recent common ancestor for the SCC*mec* type I elements in the archaic clone isolates examined is 1941.

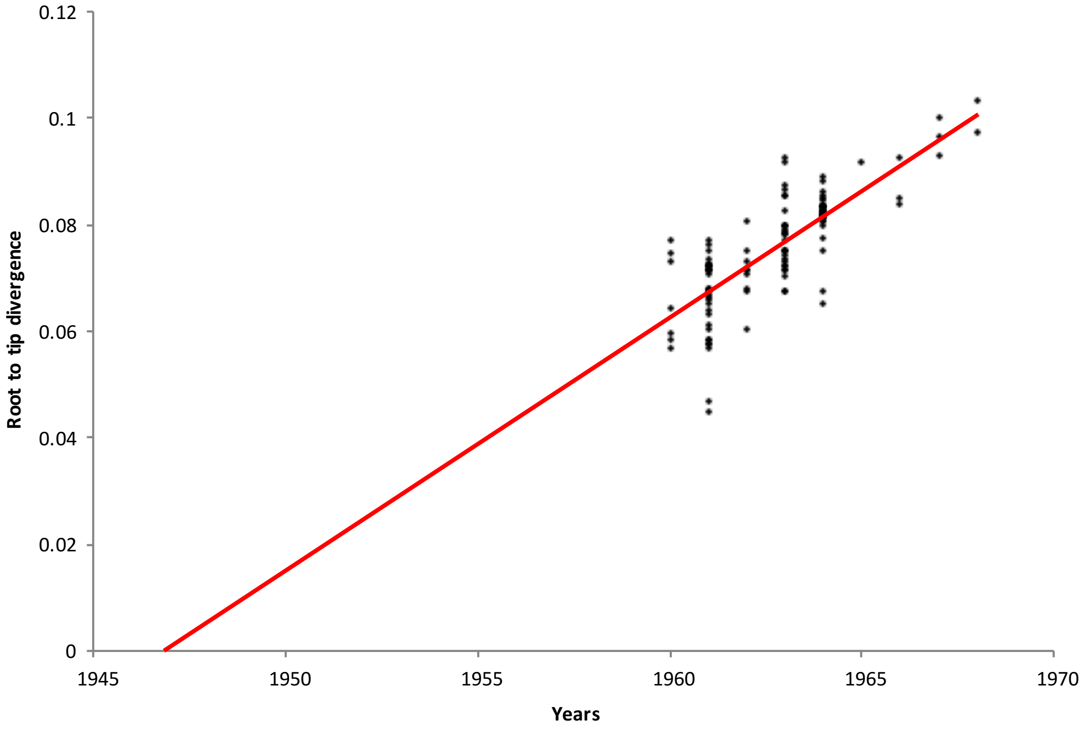

Additional file 6: Figure S4

Linear regression of the root-to-tip distances of the archetypal MRSA clone population used for BEAST analysis.

The analysis was carried out using Path-O-Gen v1.4 (http://tree.bio.ed.ac.uk/software/pathogen/) with a best-fit root from the maximum likelihood tree and the dates of isolation. The plot contains straight-line best fit of the root-to-tip divergence for each of the isolates, with a correlation coefficient of 0.7907 and an R^2 of 0.6525. The time to most recent common ancestor for the whole population was estimated to be 1947, consistent with the Bayesian analyses (as illustrated in Figure 3B).
