# Supplementary material for: Methicillin-resistant Staphylococcus aureus emerged long before the introduction of methicillin into clinical practice
Source: Genome Biol. 2017 Jul 20;18:130. doi: 10.1186/s13059-017-1252-9 (PMC5517843; doi:10.1186/s13059-017-1252-9)
Supplement: Supplementary file 4 — Resistome antibiotic determinants. Updated resistome database used for the in silico identification of antibiotic-resistant determinants based on the database previous described by Aanensen et al., 2016. Included in the database are antibiotics and associated acquired genes and core gene mutations. Accession numbers for the acquired genes and protein sequences are provided, as well as amino acid substitution information for the core genes. (PDF 80 kb) [file 13059_2017_1252_MOESM4_ESM.pdf]

Antibiotic	Accessory gene	Protein Accession	DNA Accession	Core gene	Mutation	Substitution	Reference
**Penicillin**	*blaZ*	BLAC_STAAU	CAA27733				
	blaZ-D	Q53699_STAAU	AAA26647				
	blaZ-A	Q60250_STAAU	AAA26644				
	$blaZ_{LGA251}$	F4NA56_STAAU	CBZ41939				
**Methicilin**	*mecA*	O54286_STAAU	AAQ75792				
	mecC	F4NA57_STAAU	AKP21265				
**Ciprofloxacin**				*grlA*		S80F	[1]
				grlA & *gyrA*		S80F, S84L	[1]
				grlA & *gyrA*		S80Y, S84L	[1]
				grlA & *gyrA*		S80F, E88K	[1]
				grlA		S80Y	[1]
**Moxifloxacin**				*grlA* & *gyrA*		S80F, S84L	[1]
				grlA & *gyrA*		S80Y, S84L	[1]
**Gentamicin**	*aacA-aphD*	AACA_STAAM	BAB47534				
**Amikacin**	*aphA*-3	A8J4N5_STAAU	BAF82029				
	aadD	KANU_STAAU	CAA27142				
**Tobramycin**	*aadD*	KANU_STAAU	CAA27142				
	aacA-aphD	AACA_STAAM	BAB47534				
**Streptomycin**	*str*	STR_STAAU	CAA29839				
	aad9	S3AD_STAAN	BAA82204				
	aadE	A8J4N2_STAAU	BAF82028				
**Erythromycin**				23S rRNA	A2058T/G		[2]
					A2059G		[2]
	ermA	ERMA_STAAN	BAA82205				
	ermA-like	A1E139_STAEP	ABK81677				
	ermB	Q799N2_STAAU	ABQ00061				
	ermC	ERMC3_STAAU	AAA20192				
	erm33	Q8KLN5_STASC	CAC86410				
	ermT	D3GMR8_STAA5	CAY48681				
	mefA	Q79RU3_STAAU	AAL58635				
	mefE	Q8DPW8_STRR6	AAK99775				
	msrA	A0A068LCQ4_STAAU	AIE40079				
	ereA	EREA_ECOLX	AAO38247				
	ereB	EREB_ECOLX	CAA27626				
**Clindamycin**	*linA*	Q6QHH8_STAAU	AAS50177				
	ermA	ERMA_STAAN	BAA82205				
	ermC	ERMC3_STAAU	AAA20192				
**Tetracycline**	*tetK*	TCR_STAAU	AAB07712				
	tetM	TETM_STAAM	BAB56560				
	tetL	C7C2U6_STAA5	CAY33088				
	tetO	TETO_STRMU	AAA26679				
**Fusidic acid**				*fusA*		V90I	[3]
						E444K	[4]
						G451V	[4]
						M453I	[4]

						H457Y	[4]
						L461K	[4]
						L461S	[4]
						P478S	[4]
						M651I	[4]
						P404L	[4]
						G452S	[5]
						H457Q	[6]
	fusB	Q8GNY5_STAAU	AAL12234				
	fusC	A0A0P0YP43_STAHO	BAT22900				
**Linezolid**				23S rRNA	G2447T		[7]
					T2500A		[8]
					A2503G		[9]
					T2504C		[9]
					G2505A		[10]
					G2576T		[11]
				rplD		K68Q	[7]
				rplC		G155R	[7]
						∆F127-H146	[7]
	cfr	CFR_STASC	CAC04525				
**Chloramphenicol**	*catA*1	CAT2_STAAU	AAA72572				
	*catA*2	Q6V0Y2_STAAU	AAQ55242				
	*catA*3	CAT1_STAAU	CAA24586				
**Mupirocin**				*IleS-1*		V588F	[12]
						G593V	[12]
						V631F	[12]
	ileS-2	SYI2_STAAU	CAA53189				
**Rifampicin**				*rpoB*		S464P	[13]
						Q468R	[13]
						D471Y	[13]
						A477V	[13]
						A477D	[13]
						H481Y	[13]
						R484H	[13]
						D550G	[13]
**Trimethoprim**				*dfrA*		L41F	[14]
						F99S	[14]
						F99Y	[14]
						H150R	[14]
	dfrK	C7C2U7_STAA5	CAY33090				
	dfrB	DYR_STAAM	BAB57588				
	dfrG	Q4H3Y3_STAAU	AQR07665				
**Disinfectants**	*qacA*	QACA_STAAM	BAB47540				
	qacC	QACC_STAAU	AAA26666				
	qacG	QACG_STAS9	CAA76542				
	qacH	QACH_STASA	CAA76544				

qacJ	Q84EL2_STAAU	CAD55144

References

1. Griggs DJ, Marona H, Piddock LJV. Selection of moxifloxacin-resistant *Staphylococcus aureus* compared with five other fluoroquinolones. J. Antimicrob. Chemother. Oxford University Press; 2003;51:1403–7.

2. Prunier A-L, Malbruny B, Laurans M, Brouard J, Duhamel J-F, Leclercq R. High rate of macrolide resistance in *Staphylococcus aureus* strains from patients with cystic fibrosis reveals high proportions of hypermutable strains. J. Infect. Dis. Oxford University Press; 2003;187:1709–16.

3. Lannergård J, Norström T, Hughes D. Genetic determinants of resistance to fusidic acid among clinical bacteremia isolates of *Staphylococcus aureus*. Antimicrob. Agents Chemother. American Society for Microbiology; 2009;53:2059–65.

4. Chen H-J, Hung W-C, Tseng S-P, Tsai J-C, Hsueh P-R, Teng L-J. Fusidic acid resistance determinants in *Staphylococcus aureus* clinical isolates. Antimicrob. Agents Chemother. American Society for Microbiology; 2010;54:4985–91.

5. Besier S, Ludwig A, Brade V, Wichelhaus TA. Molecular analysis of fusidic acid resistance in *Staphylococcus aureus*. Mol Microbiol. 2003;47:463–9.

6. Castanheira M, Watters AA, Mendes RE, Farrell DJ, Jones RN. Occurrence and molecular characterization of fusidic acid resistance mechanisms among *Staphylococcus* spp. from European countries (2008). J. Antimicrob. Chemother. Oxford University Press; 2010;65:1353–8.

7. Locke JB, Hilgers M, Shaw KJ. Novel ribosomal mutations in *Staphylococcus aureus* strains identified through selection with the oxazolidinones linezolid and torezolid (TR-700). Antimicrob. Agents Chemother. American Society for Microbiology; 2009;53:5265–74.

8. Meka VG, Pillai SK, Sakoulas G, Wennersten C, Venkataraman L, DeGirolami PC, et al. Linezolid resistance in sequential *Staphylococcus aureus* isolates associated with a T2500A mutation in the 23S rRNA gene and loss of a single copy of rRNA. J. Infect. Dis. Oxford University Press; 2004;190:311–7.

9. Livermore DM, Warner M, Mushtaq S, North S, Woodford N. *In vitro* activity of the oxazolidinone RWJ-416457 against linezolid-resistant and -susceptible staphylococci and enterococci. Antimicrob. Agents Chemother. American Society for Microbiology; 2007;51:1112–4.

10. Bourgeois-Nicolaos N, Massias L, Couson B, Butel M-J, Andremont A, Doucet-Populaire F. Dose dependence of emergence of resistance to linezolid in *Enterococcus faecalis in vivo*. J. Infect. Dis. 2007;195:1480–8.

11. Tsiodras S, Gold HS, Sakoulas G, Eliopoulos GM, Wennersten C, Venkataraman L, et al. Linezolid resistance in a clinical isolate of *Staphylococcus aureus*. Lancet. 2001;358:207–8.

12. Hurdle JG, O'Neill AJ, Ingham E, Fishwick CWG, Chopra I. Analysis of mupirocin resistance and fitness in *Staphylococcus aureus* by molecular genetic and structural modeling techniques. Antimicrob. Agents Chemother. American Society for Microbiology; 2004;48:4366–76.

13. Aubry-Damon H, Soussy CJ, Courvalin P. Characterization of mutations in the *rpoB* gene that confer rifampin resistance in *Staphylococcus aureus*. Antimicrob. Agents Chemother. 1998;42:2590–4.

14. Vickers AA, Potter NJ, Fishwick CWG, Chopra I, O'Neill AJ. Analysis of mutational resistance to trimethoprim in *Staphylococcus aureus* by genetic and structural modelling techniques. J. Antimicrob. Chemother. Oxford University Press; 2009;63:1112–7.
